# Mediating Role of the ANGPTL3/TFPI Protein Ratio in Regulating T-Cell Surface Glycoprotein CD5 Levels on Knee Osteoarthritis (KOA): A Mendelian Randomization Study

**DOI:** 10.3390/ijms26104471

**Published:** 2025-05-08

**Authors:** Yongwei Li, Xi Liao, Xi Yu, Ying Xiao, Xiaoyu Tao, Tian Zhong

**Affiliations:** Faculty of Medicine, Macau University of Science and Technology, Taipa, Macao 999078, Chinamust.derek@outlook.com (X.T.)

**Keywords:** inflammatory proteins, knee osteoarthritis (KOA), Mendelian randomization (MR), ratios between protein levels (rQTLs)

## Abstract

This study utilized Mendelian randomization (MR) to investigate the impact of inflammatory proteins on knee osteoarthritis (KOA), measured using the ratio of protein levels (rQTLs). The primary objective was to identify potential intervention targets to mitigate KOA progression. Data from 2821 rQTLs, 91 inflammatory proteins, and KOA-related genetic variations were obtained through genome-wide association studies (GWAS). Bidirectional MR identified rQTLs with unidirectional causal relationships with KOA. Further analyses included false discovery rate (FDR) correction, colocalization, and mediation analysis. Two inflammatory proteins were found to be associated with KOA: T-cell surface glycoprotein CD5 [OR (95% CI) = 0.867 (0.760–0.990), *P*_IVW_ = 0.035] and C-X-C motif chemokine 9 [OR (95% CI) = 1.150 (1.001–1.320), *P*_IVW_ = 0.048]. Variations in their levels influenced rQTLs, producing differential effects on KOA. Specifically, rQTL-ANGPTL3/TFPI (human recombinant angiopoietin-like protein 3/Tissue factor pathway inhibitor) was identified as a mediator in the effect of T-cell surface glycoprotein CD5 levels on KOA. T-cell surface glycoprotein CD5 levels were negatively correlated with rQTL-ANGPTL3/TFPI (*β*1 = −0.084), while rQTL-ANGPTL3/TFPI was positively correlated with KOA (*β*2 = 0.159). These findings align with the total effect, where T-cell surface glycoprotein CD5 levels were negatively associated with KOA (*β* = −0.143). Thus, rQTL-ANGPTL3/TFPI may serve as a reliable mediator in the pathway through which T-cell surface glycoprotein CD5 levels affect KOA. This mediator may not only represent a potential therapeutic target but also serve as a biomarker for assessing KOA treatment efficacy, offering a novel direction for KOA diagnosis and management.

## 1. Introduction

Knee osteoarthritis (KOA) is a progressive chronic disorder characterized by degenerative alterations in joint cartilage, subchondral bone, ligaments, joint capsules, synovium, and periarticular muscles [1,2]. It constitutes 83% of the overall burden of arthritis [3]. As of 2020, approximately 65.4 million individuals worldwide were affected by KOA [4], with 20% of those aged 65 and above experiencing symptomatic KOA. While traditionally regarded as an age-related condition, recent evidence indicates that the prevalence of KOA is on the rise among younger populations [4,5].

Currently, age, gender, body mass index (BMI), and imaging severity are commonly used to predict the progression of KOA. In addition, factors such as overuse in occupational activities and sports, musculoskeletal injuries, and obesity have all been confirmed as significant contributing causes to the occurrence and development of KOA [6,7]. However, these indicators primarily provide a superficial estimation of the disease and are limited to initial structural assessments. They lack the capability to offer detailed predictions regarding subsequent disease progression and clinical outcomes [8]. The primary reason is that the onset and progression of osteoarthritis involve dynamic changes mediated by inflammatory factors, cytokines, chemokines, growth factors, and adipokines. In theory, it is possible to achieve more precise predictions of disease progression by monitoring biomarkers in serum, synovial fluid, and histological samples. For instance, changes in the levels of C-terminal telopeptide of type II collagen (CTX-II), a degradation product of type II collagen present in the urine, can serve as a reliable and sensitive biomarker for monitoring the progression of cartilage degradation [9].

Aging chondrocytes disrupt the cellular redox homeostasis [10] and secrete pro-inflammatory factors, including p16 (p16 inhibitor of CDK4), CDK2 (cyclin-dependent kinase 2), Cyclin D1, IL-6 (Interleukin-6), IL-1β (Interleukin-1β), and TNF-α (Tumor Necrosis Factor-α) [11,12]. These factors activate relevant signaling pathways, thereby inducing inflammatory responses. For example, retinoic acid receptor-related orphan receptor α (RORα) downregulates the expression of cartilage matrix components such as type II collagen and aggrecan while upregulating the IL-6/STAT3 (Interleukin-6/Signal Transducer and Activator of Transcription 3) signaling pathway [13]. Furthermore, damaged chondrocytes produce excessive reactive oxygen species (ROS), leading to apoptosis and the activation of pro-inflammatory responses, which exacerbate the development of KOA. Autophagy, acting as a protective mechanism under inflammatory conditions, mitigates KOA by upregulating anti-inflammatory proteins such as IL-10 (Interleukin-10), reducing synovial inflammation and the degradation of cartilage proteoglycans [14]. However, as the activity of autophagy-derived anti-inflammatory proteins gradually declines, the capacity to clear damaged tissues weakens [15], resulting in the accumulation of oxidized protein products, such as Fap (fetuin-A), CRLF1 (cytokine receptor-like factor 1), GLG1 (galectin-1), and CSPG4 (chondroitin sulfate proteoglycan 4) [8,16,17]. This accumulation activates SASP (senescence-associated secretory phenotype) factors, such as matrix metalloproteinase MMP-13 (matrix metalloproteinase-13) [18], thereby promoting articular cartilage degeneration. Notably, not all protein products accelerate KOA progression. Under specific conditions, the activation of pro-inflammatory proteins such as GPCRs (G-Protein-Coupled Receptors) [19] can maintain the balance between cartilage matrix synthesis and degradation, preserve cartilage homeostasis, and slow the progression of KOA.

Inflammation-related proteins can induce alterations in the levels of various protein products within the body [20,21,22], thereby influencing the onset and progression of KOA. These protein products play critical roles in inflammatory responses, immune regulation, and tissue damage. Moreover, protein ratios reflect the relative levels and interactions between different proteins, offering key insights into the complex mechanisms underlying KOA. Given the current paucity of research on the influence of rQTLs on KOA, this study introduces the concept of rQTLs, hypothesizing that rQTLs serve as intermediate variables through which inflammation-related proteins impact KOA. We have attempted to further investigate the potential role of rQTLs in the pathogenesis of KOA using MR analysis.

## 2. Results

The inflammatory proteins and rQTLs associated with KOA were identified.

**Figure 1:**
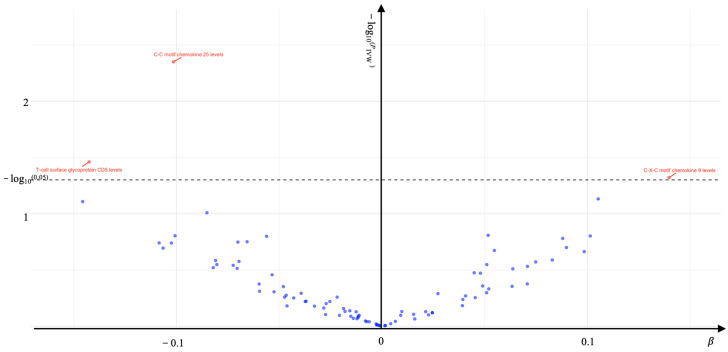
Associations of 91 inflammatory protein genes with KOA. The associations that survived multiple testing rounds are labeled in the volcano plot. Red dots above the dashed line represent significant associations, blue dots below the dashed line represent insignificant associations.

In the forward MR analysis, causal genes associated with KOA were identified as the outcome, with processed inflammatory protein levels used as the exposure. The IVW method confirmed that three inflammatory proteins are causally related to KOA (*P*_IVW_ < 0.05) (Figure 1). Additionally, reverse MR analysis showed no evidence of reverse causality (Reverse MR *P*_IVW_ > 0.05) (Table 1), indicating a unidirectional effect of inflammatory proteins on KOA. Specifically, the C-C motif chemokine 25 levels [OR (95%*CI*) = 0.904 (0.843–0.969) *P*_IVW_ = 0.004] and T-cell surface glycoprotein CD5 levels [OR (95%*CI*) = 0.867 (0.760–0.990) *P*_IVW_ = 0.035] were associated with a protective effect against KOA, while C-X-C motif chemokine 9 levels [OR (95%*CI*) = 1.150 (1.001–1.320) *P*_IVW_ = 0.048] were associated with an increased risk of developing KOA (Table 1).

**Figure 2:**
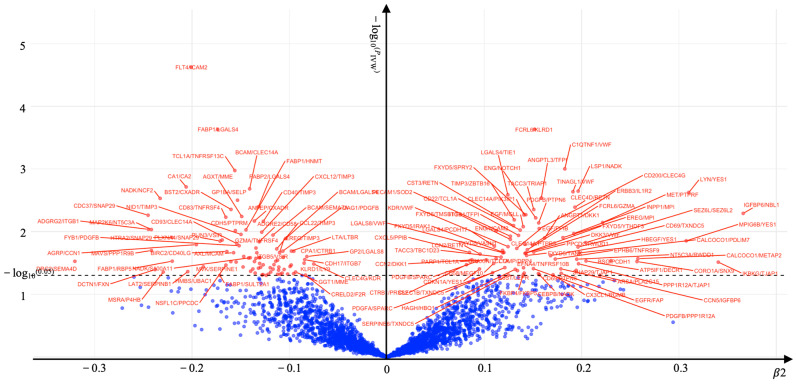
Associations of 2821 rQTL genes with KOA. The associations that survived multiple testing are labeled in the volcano plot. Red dots above the dashed line represent significant associations, blue dots below the dashed line represent insignificant associations.

In the forward MR analysis, KOA-associated genes were identified as the outcome, while processed rQTLs served as the exposure. Using the IVW method, we confirmed that 147 rQTLs had a causal relationship with KOA (*P*_IVW_ < 0.05) (Figure 2). Subsequently, 147 rQTLs were used as the outcome in the reverse MR analysis, with KOA serving as the exposure. Nine rQTLs demonstrated reverse causality (*P*_IVW_ < 0.05) (Table 2). Therefore, these nine rQTLs were excluded from the subsequent analysis investigating the relationship between inflammatory proteins and rQTLs.

**Figure 3:**
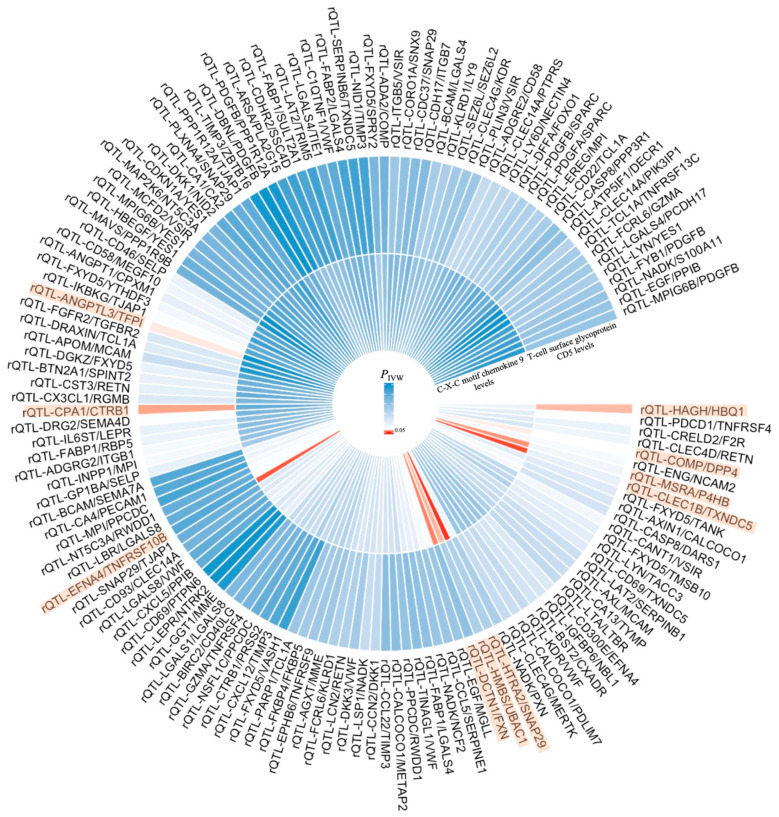
Causal relationships between inflammatory proteins and rQTLs associated with KOA. Out of 2821 rQTLs, 138 were identified to have unidirectional causal associations with KOA. Among 91 inflammatory proteins, two exhibited such associations with KOA. A bidirectional MR analysis was performed, generating a circular heatmap of the causal relationships. The inner circle represents C-X-C motif chemokine 9 levels, the middle circle represents T-cell surface glycoprotein CD5 levels, and the outer circle represents the 138 rQTLs. Red indicates a significant causal relationship (*P*_IVW_ < 0.05), while blue indicates no significant causal relationship (*P*_IVW_ > 0.05).

**Figure 4:**
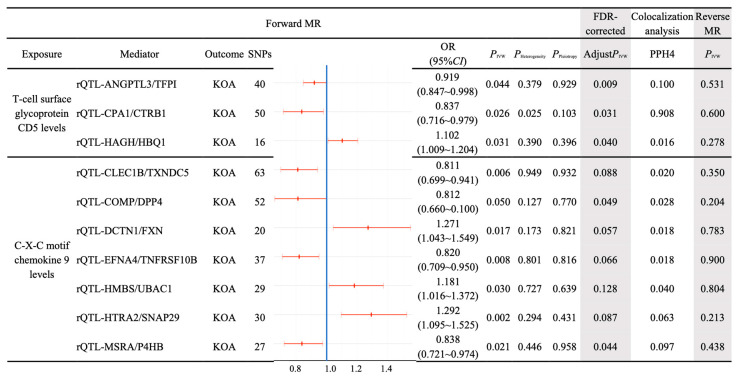
Causal analysis of exposures and mediator rQTLs associated with KOA. Two exposures significantly associated with KOA were grouped, and mediator rQTLs with unidirectional causal associations were selected. A dendrogram illustrates the odds ratio (OR) for these rQTLs. Subsequent analyses included FDR correction, colocalization, and reverse MR.

We conducted a bidirectional MR analysis using genetic variants associated with three selected inflammatory proteins as exposures and the 138 rQTLs as outcomes. In the MR analysis for C-C motif chemokine 25 levels, only one SNP (*rs34520165*) remained, indicating a high possibility of error in conducting causal analysis. Therefore, C-C motif chemokine 25 levels were excluded, and the pathogenic genes of the remaining two inflammatory proteins were analyzed as exposures (Figure 3). The IVW method confirmed that the pathogenic genes of T-cell surface glycoprotein CD5 levels had a causal relationship with three rQTLs (ANGPTL3/TFPI, CPA1/CTRB1, HAGH/HBQ1), and the pathogenic genes of C-X-C motif chemokine 9 levels had a causal relationship with seven rQTLs (CLEC1B/TXNDC5, COMP/DPP4, DCTN1/FXN, EFNA4/TNFRSF10B, HMBS/UBAC1, HTRA2/SNAP29, MSRA/P4HB). Separate reverse MR analyses indicated the absence of reverse causality (reverse MR *P*_IVW_ > 0.05) (Figure 4). Therefore, the causal relationship in this conclusion is unidirectional, and the changes in inflammatory proteins result in corresponding variations in rQTLs.

**Figure 5:**
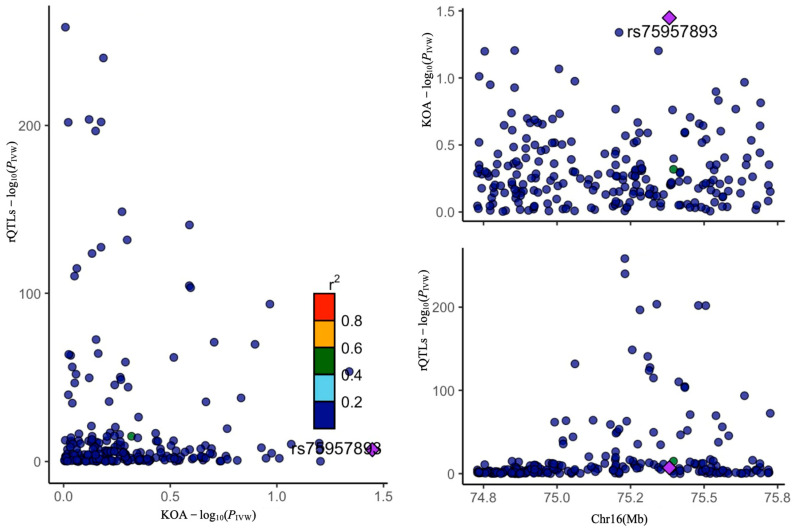
rQTL-CPA1/CTRB1 colocalization analysis with KOA.

Bayesian colocalization analyses were performed for each of the 10 selected rQTLs (ANGPTL3/TFPI, CPA1/CTRB1, HAGH/HBQ1, CLEC1B/TXNDC5, COMP/DPP4, DCTN1/FXN, EFNA4/TNFRSF10B, HMBS/UBAC1, HTRA2/SNAP29, MSRA/P4HB) in relation to KOA. The results demonstrated that only the causal relationship between rQTL-CPA1/CTRB1 and KOA was relatively stable (PPH4 = 0.908 > 0.75). Despite the presence of horizontal pleiotropy in rQTL-CPA1/CTRB1 (PPleiotropy = 0.025) (Figure 4), the findings remained credible (Figure 5). For the remaining nine rQTLs (PPH4 < 0.75), their potential causal relationships with KOA could not be ruled out (Figure 4). Since colocalization analysis cannot fully account for confounding factors such as gene–race and gene–environment interactions, its applicability to other populations is restricted. The rationale for retaining the colocalization conclusion lies in its continued relevance for the European population. By integrating colocalization with MR analysis, the conclusions remain robustly supported within the European context.

The MR analysis of three rQTLs (ANGPTL3/TFPI, CPA1/CTRB1, HAGH/HBQ1) influenced by T-cell surface glycoprotein CD5 levels on KOA, after FDR correction, yielded statistically significant results (Adjust*P*_IVW_ < 0.05). Among the seven rQTLs influenced by C-X-C motif chemokine 9 levels on KOA, only two rQTLs (COMP/DPP4 and MSRA/P4HB) remained statistically significant (Adjust*P*_IVW_ < 0.05) (Figure 4).

Three screened rQTLs (ANGPTL3/TFPI, CPA1/CTRB1, HAGH/HBQ1) were, respectively, employed as the mediator variables between T-cell surface glycoprotein CD5 levels and KOA. The mediating effect of ANGPTL3/TFPI (mediated proportion = 9.340%) was consistent with the total effect and participated in improving KOA, while the mediating effects of CPA1/CTRB1 (mediated proportion = −11.825%) and HAGH/HBQ1 (mediated proportion = −8.954%) were contrary to the total effects and would aggravated the occurrence of KOA (Table 3).

Two screened rQTLs (COMP/DPP4, MSRA/P4HB) were, respectively, used as the mediator variables between C-X-C motif chemokine 9 levels and KOA. The mediating effect of COMP/DPP4 (mediated proportion = −19.909%) was contrary to the total effect and contributed to the improvement of KOA. The mediating effect of MSRA/P4HB (mediated proportion = 29.079%) was consistent with the total effect and contributed to the aggravation of the occurrence of KOA (Table 3).

## 3. Discussion

KOA is a chronic inflammatory disease. Current research demonstrates that T cells play a critical role in the pathogenesis of KOA. Specifically, Th1, Th9, and Th17 subtypes contribute to the exacerbation of KOA, whereas the effects of Th2, Th22, Tfh, and unconventional T cells on KOA remain unclear [23]. Moreover, Treg cells mitigate KOA progression by inhibiting osteoclast activity [23,24]. Concurrently, the release of cytotoxic chemokines disrupts cartilage homeostasis, leading to cartilage degradation and further aggravating KOA development [23]. These pathological changes induce dynamic alterations in plasma protein levels, which in turn influence the onset and progression of KOA. Therefore, the relative changes in plasma protein ratios more accurately reflect the key pathological processes involved in KOA. In this study, plasma protein ratios are employed as intermediate variables reflecting the impact of inflammatory proteins on KOA. This approach not only identifies potential intervention targets for KOA treatment but also provides biomarkers for evaluating therapeutic efficacy and prognosis. Thus, this approach holds great significance for the precise management and prognostic assessment of KOA.

This study performed MR analysis and colocalization analysis based on the European population. However, given that the study subjects were restricted to Europeans, it remains challenging to entirely exclude the potential influence of gene–race confounding factors on the conclusions. Consequently, the extrapolation of findings derived from combining MR and colocalization analyses to other races may be constrained. The rQTLs mediation effect identified is highly reliable within the European population but requires further validation for its applicability in other populations. In contrast, the biological function of individual proteins can be determined through meticulously designed experiments, effectively minimizing interference from confounding factors such as heterogeneity, pleiotropy, gene–race interactions, and gene–environment interactions. Integrating this approach with MR analysis not only addresses the limitations associated with the European population but also enhances the generalizability and robustness of the conclusions. This ensures the scientific validity of the rQTLs obtained in a broader population and provides a solid foundation for subsequent cellular and animal model studies. Therefore, the following discussion will focus on elucidating the impact of proteins on KOA via their biological functions while leveraging the identified rQTLs as mediating variables to comprehensively analyze the mechanisms underlying the effects of inflammatory proteins on KOA, aiming to achieve consistent and universally applicable conclusions.

### 3.1. The Impact of T-Cell Surface Glycoprotein CD5 Levels on KOA

#### 3.1.1. The Total Effect (T-Cell Surface Glycoprotein CD5 Levels Were Negatively Correlated with KOA (*β* = −0.143))

T cells and their surface glycoproteins play a pivotal role in the onset and progression of KOA. As a key component of the immune system, T cells modulate and drive the joint immune response by releasing multiple cytokines, chemokines, and inflammatory mediators, thereby actively contributing to the pathological process of KOA [25]. CD5 is a type I transmembrane glycoprotein expressed on the surface of T cells. Proteomic analyses have demonstrated that CD5 exhibits remarkable specificity in the synovial fluid of patients with rheumatoid arthritis (RA) and KOA. Notably, CD5 expression is significantly upregulated in the synovial fluid of KOA patients, indicating its potential involvement in OA-associated inflammatory processes. This observation not only underscores the relevance of CD5 in both RA and KOA but also reinforces its likely role in immune regulation and synovial joint inflammation [26]. The presence of CD5 promotes T cell activation, interleukin-2 (IL-2) production, and the differentiation of pathogenic Th17 cells [27]. Furthermore, upon activation of effector T cells, the TRPC1-mediated B cell receptor (BCR)-independent calcium signaling pathway enhances the generation of the anti-inflammatory cytokine IL-10 [28], effectively mitigating the symptoms of KOA. An animal study involving the injection of IL-10 protein into the knee joints of mice and subsequent analysis of whole-genome expression in arthritic synovial tissue confirmed that IL-10 overexpression not only significantly alleviates synovitis but also markedly reduces the loss of cartilage proteoglycans, further validating this mechanism [14]. Therefore, this study demonstrates that the level of the T cell surface glycoprotein CD5 is inversely correlated with the severity of KOA.

#### 3.1.2. The Mediating Effect of rQTL-ANGPTL3/TFPI

This research found that the levels of T-cell surface glycoprotein CD5 were negatively correlated with rQTL-ANGPTL3/TFPI (*β*1 = −0.084); rQTL-ANGPTL3/TFPI was positively correlated with KOA (*β*2 = 0.159). When the levels of T-cell surface glycoprotein CD5 increased, the mediating variable rQTL-ANGPTL3/TFPI decreased, thereby mitigating KOA, which was consistent with the overall effect.

Researchers successfully established an *in vitro* cell model by treating cultured THP-1-derived macrophages with human recombinant angiopoietin-like protein 3 (ANGPTL3), inducing their polarization into a pro-inflammatory phenotype. The results demonstrated that ANGPTL3 significantly upregulated the expression of inflammatory factors, including interleukin-1β (IL-1β), interleukin-6 (IL-6), and tumor necrosis factor α (TNFα), in differentiated THP-1 macrophages via its glycosylation and proteolytic cleavage functions [29]. Furthermore, genetic studies and mouse animal model experiments revealed that circulating ANGPTL3 protein inhibits lipoprotein lipase (LPL) activity, leading to elevated levels of triglycerides, low-density lipoprotein cholesterol (LDL-C), high-density lipoprotein cholesterol (HDL-C), and total cholesterol [30]. This metabolic disturbance subsequently triggers increased reactive oxygen species (ROS) production, amplifies chondrocyte inflammation, enhances extracellular matrix (ECM) catabolism, and induces chondrocyte apoptosis. At the same time, it suppresses LRP3 gene expression in chondrocytes, thereby adversely affecting ECM metabolism and exacerbating cartilage degradation [31], which may ultimately contribute to the development of KOA. On the other hand, ANGPTL3 plays a critical role in maintaining the stemness of hematopoietic stem cell (HSC) niches, and osteoclast localization is closely associated with HSC retention within these niches. Therefore, reducing Angptl3 levels can effectively decrease osteoclast expression, offering a potential therapeutic strategy for improving KOA [32].

Tissue factor pathway inhibitor (TFPI) is an anticoagulant glycoprotein continuously synthesized and secreted by the vascular wall of the microvascular system [33] and belongs to the serine protease family [34]. Human control experiments and mouse studies have demonstrated that thrombosis in the microcirculation of bone marrow microvessels inhibits osteocyte activity and diminishes osteoblast remodeling capacity. Through the positive feedback mechanism of heparanase, the synergistic upregulation of TFPI expression can restore hemostatic balance and enhance osteoblast activity, thereby improving bone tissue repair capability, which may facilitate arthritis repair [35]. Additionally, another human control experiment revealed that persistent fibrin deposition induces arthritis and that TFPI levels in synovial fluid are significantly lower than those in healthy individuals. Given that TFPI, as a fibrinolytic factor, can inhibit fibrin formation and alleviate arthritis symptoms, increasing TFPI expression may positively contribute to the improvement of knee arthritis [36].

From a biological standpoint, studies have demonstrated that reducing the levels of rQTL-ANGPTL3/TFPI is likely to positively influence the improvement of KOA. Furthermore, when rQTL-ANGPTL3/TFPI serves as a mediator in the regulation of KOA via T-cell surface glycoprotein CD5 levels, its effect aligns with the total effect observed.

#### 3.1.3. The Mediating Effect of rQTL-CPA1/CTRB1

This research discovered that the levels of T-cell surface glycoprotein CD5 were negatively correlated with rQTL-CPA1/CTRB1 (*β*1 = −0.178), and rQTL-CPA1/CTRB1 was negatively correlated with KOA (*β*2 = −0.095). When the levels of T-cell surface glycoprotein CD5 increased, the mediating variable rQTL-CPA1/CTRB1 decreased, exacerbating KOA. The colocalization analysis strengthened the evidence for the influence of rQTL-CPA1/CTRB1 on KOA. Theoretically, blocking the decrease in the mediating variable rQTL-CPA1/CTRB1 could alleviate KOA. This was inconsistent with the overall effect.

Researchers utilizing MR analysis conducted a controlled study involving 103 patients with radiologically confirmed KOA and 86 healthy controls. The aim was to measure resistin levels in serum and synovial fluid and evaluate its effects on chondrocytes and cyclic adenosine monophosphate (cAMP) phosphodiesterase 1 (CAP1) [37]. Furthermore, by treating mouse chondrocyte cultures with recombinant resistin, the inhibitory effect of CAP1 on proteoglycan synthesis in human chondrocyte explants was examined [38]. The findings demonstrate that CAP1 in serum and synovial fluid primarily binds to resistin (an adipokine), thereby activating signaling pathways in chondrocytes, such as nuclear factor κB (NF-κB) and p38 mitogen-activated protein kinase (p38-MAPK). This activation induces pro-inflammatory cytokine production and matrix-degrading enzyme activity while downregulating the expression of structural proteins in the extracellular matrix of articular chondrocytes [38], ultimately contributing to the progression of KOA.

Chymotrypsinogen B1 (CTRB1) is a member of the serine protease family. MR studies of type 1 diabetes (T1D) patients (9684 T1D patients and 15,743 controls) have shown that CTRB1 plays a significant role in improving T1D through digestive enzyme secretion [39]. Furthermore, a clinical study involving eight T1D patients without a history of knee trauma and nine healthy volunteers confirmed that T1D can lead to KOA by altering cartilage metabolism. Therefore, CTRB1 may reduce KOA associated with T1D by regulating cellular metabolism, including insulin synthesis and digestive enzyme secretion [40]. From a biological perspective, the reduction in rQTL-CPA1/CTRB1 appears beneficial for improving KOA.

Therefore, from a biological perspective, the reduction in the rQTL-CPA1/CTRB1 ratio may exacerbate KOA, which is inconsistent with the direction of the total effect. Specifically, when rQTL-CPA1/CTRB1 serves as a mediator in the regulation of KOA by T-cell surface glycoprotein CD5 levels, its effect direction contradicts the total effect. This phenomenon may arise because the decreased rQTL-CPA1/CTRB1 ratio compromises the compensatory mechanism, thereby weakening the protective role of CTRB1 and failing to sufficiently suppress the detrimental effects of CPA1, thus aggravating KOA progression. Moreover, the indirect exacerbation of KOA due to reduced rQTL-CPA1/CTRB1 ratios coexists with the direct ameliorative effects of T-cell surface glycoprotein CD5 levels; however, the presence of rQLTs with opposing regulatory effects partially offsets the mediating role of rQTL-CPA1/CTRB1, further driving KOA progression. Finally, when CPA1 and CTRB1 jointly influence KOA, new regulatory factors may emerge due to the incomplete characterization of their complex interactions. The interplay among these factors may lead to an underestimation of the total effect as a singular outcome, whereas multiple effects exist, ultimately reflecting the worsening trend of KOA.

#### 3.1.4. The Mediating Effect of rQTL-HAGH/HBQ1

This study revealed that the levels of T-cell surface glycoprotein CD5 were positively correlated with rQTL-HAGH/HBQ1 (*β*1 = 0.097), and rQTL-HAGH/HBQ1 was positively correlated with KOA (*β*2 = 0.132). When the levels of T-cell surface glycoprotein CD5 increased, the mediating variable rQTL-HAGH/HBQ1 rose, aggravating KOA. Theoretically, blocking the increase in the mediating variable rQTL-CPA1/CTRB1 could alleviate KOA, which was inconsistent with the total effect.

Current research indicates that Hydroxyacylglutathione hydrolase (HAGH/GLO2) and GLO1 together constitute the glyoxalase system, which protects cells from glycation damage during the elimination of methylglyoxal. The normal function of these enzymes is crucial for cellular antioxidant capacity and resistance to glycation reactions [41]. Under the synergistic effect of the p53-p21 axis, GLO2 can reduce oxidative stress responses, thereby alleviating p53 activation and promoting cell proliferation. Additionally, it helps cells evade apoptosis by reducing cellular oxidative damage and inhibiting p53-mediated apoptotic signals. Therefore, through its synergy with the p53-p21 axis, GLO2 not only promotes cell proliferation but also assists cells in evading apoptosis, potentially improving KOA. This mechanism was validated in a study that analyzed the selective expression of GLO2 in 20 prostate tumor cell cases using immunohistochemistry [42].

Animal model experiments have demonstrated that hemoglobin subunit θ1 (HBQ1) is expressed in red blood cells and plays a role in oxygen transport [43]. The high-level expression of HBQ1 effectively decreases basal ROS levels, mitigates oxidative damage induced by elevated ROS [44], thereby enhancing cell growth and proliferation, and may contribute positively to the improvement of KOA [45].

From the perspective of individual plasma proteins, HAGH and HBQ1 may potentially confer benefits for KOA. However, the precise role of rQTL-HAGH/HBQ1 remains elusive, and its potential influence on KOA warrants further investigation. Therefore, it is essential to conduct in-depth research to ascertain whether rQTL-HAGH/HBQ1 serves as a mediator in the regulation of KOA. This study further reveals that when rQTL-HAGH/HBQ1 mediates the regulation of KOA by T-cell surface glycoprotein CD5 levels, its effect contradicts the total effect. The underlying reason may be that when T-cell surface glycoprotein CD5 levels rise, although the increased rQTL-HAGH/HBQ1 ratio indirectly enhances the biological functions of individual proteins, theoretically improving KOA, the disruption of the original balance leads to the over-activation of HAGH and HBQ1 protein functions. This generates conflicting signals of cartilage metabolic imbalance, resulting in compensatory mechanism failure and further exacerbating KOA progression.

### 3.2. The Impact of C-X-C Motif Chemokine 9 Levels on KOA

#### 3.2.1. The Total Effect (C-X-C Motif Chemokine 9 Levels Were Positively Correlated with KOA (*β* = 0.140))

C-X-C motif chemokines regulate immune cell migration, promote inflammatory responses, and influence the joint repair process by binding to specific CXCR receptors, thereby contributing to the pathogenesis of KOA [46]. C-X-C motif chemokine 9, a cytotoxic chemokine, serves as a key inflammatory mediator that activates Th1 cells and macrophages. A study utilized flow cytometry to detect intracellular signaling molecules, revealing the underlying immunopathological mechanisms of C-X-C motif chemokine 9 in rheumatoid arthritis (RA). The findings indicate that C-X-C motif chemokine 9 induces cartilage degradation by stimulating fibroblast-like synoviocytes (FLS) to release matrix metalloproteinases (MMPs) and other pro-inflammatory cytokines [47]. Moreover, C-X-C motif chemokine 9 facilitates the recruitment of various inflammatory cells, such as CD4+ Th1, CD8+ T, NK, and NKT cells, to the inflammatory site, leading to bone loss [48]. A retrospective analysis further demonstrated that during chemokine-mediated T-cell recruitment, C-X-C motif chemokine 9 promotes osteoclastogenesis via the IFN-γ signaling pathway, exacerbating bone destruction at the inflammatory site [49], thereby worsening the progression of KOA.

#### 3.2.2. The Mediating Effect of rQTL-MSRA/P4HB

This study discovered that C-X-C motif chemokine 9 levels were negatively correlated with rQTL-MSRA/P4HB (*β*1 = −0.177), and rQTL-MSRA/P4HB was negatively correlated with KOA (*β*2 = −0.230). When the levels of C-X-C motif chemokine 9 rose, the mediator variable rQTL-MSRA/P4HB decreased, thereby aggravating KOA. This conclusion was consistent with the total effect.

Studies have shown that the reaction mechanism of methionine sulfoxide reductase A (MSRA) overexpression in mouse liver reveals that the recombinant MSRA protein acts as an antioxidant enzyme regulated through the ROS/mitogen-activated protein kinase (MAPKs)/nuclear factor κB (NF-κB) pathway [50]. Additionally, another study observed the effects of co-administering DPCPX (a selective antagonist of the adenosine A1 receptor) and magnesium on a depression model in mice and found that MSRA has the ability to repair various oxidatively damaged proteins and prevent irreversible damage. Therefore, MSRA plays a significant role in the antioxidant stress response. When this protein is highly expressed, the lifespan of cells in an oxidative stress environment can be significantly prolonged [51]. In a cuprizone-induced demyelination mouse model, MSRA has been proven to inhibit inflammatory responses and demyelination caused by oxidative stress; conversely, the absence of MSRA in cells leads to an increase in reactive oxygen species generation within mitochondria, thereby triggering cellular oxidative damage and cell death. Thus, in addition to repairing oxidatively damaged proteins and extending cell lifespan, MSRA can also eliminate reactive oxygen species and protect the plasma membrane from lipid peroxidation damage [52], which may help improve KOA.

Recent chemical proteomic studies have revealed that, in the investigation of saturated targets of FIPC-1, prolyl 4-hydroxylase subunit β (P4HB), a member of the protein disulfide isomerase (PDI) family, has emerged as a previously unrecognized regulator of ferroptosis [53]. KOA is closely related to the process of ferroptosis. Research has confirmed that chondrocytes undergo ferroptosis under iron overload conditions, leading to increased MMP13 expression and decreased collagen II expression, which exacerbates osteoarthritis. Therefore, blocking ferroptosis can alleviate the occurrence and development of osteoarthritis [54,55]. Moreover, P4HB is negatively correlated with pro-inflammatory factors such as B cells, CD4+ T cells, CD8+ T cells, neutrophils, and macrophages [56], and its immunosuppressive and anti-inflammatory effects can improve KOA, a conclusion that was verified in an in vitro experiment [56].

Clearly, both MSRA and P4HB plasma proteins have positive effects on KOA. However, the impact of rQTL-MSRA/P4HB alterations on KOA remains uncertain and requires further investigation. When the relative proportion of MSRA decreases or the P4HB increase is too large, it leads to an imbalance in the rQTL-MSRA/P4HB ratio, making it difficult to use this ratio as a mediator to assess KOA.

#### 3.2.3. The Mediating Effect of rQTL-COMP/DPP4

This study discovered that the levels of C-X-C motif chemokine 9 were negatively correlated with rQTL-COMP/DPP4 (*β*1 = −0.208), and rQTL-COMP/DPP4 was positively correlated with KOA (*β*2 = 0.134). When the levels of C-X-C motif chemokine 9 rose, the mediator variable COMP/DPP4 decreased, thereby alleviating KOA. Theoretically, a decrease in the mediator variable rQTL-COMP/DPP4 would be beneficial for KOA. This finding is inconsistent with the total effect.

Cartilage oligomeric matrix protein (COMP) is a member of the thrombospondin family secreted by chondrocytes and is widely distributed in articular cartilage, cruciate ligaments, menisci, and tendons. This finding has been confirmed through Western blot analysis using anti-canine COMP antibodies [57]. COMP promotes cartilage tissue formation and slows the degradation of cartilage tissue in the extracellular matrix by binding to matrix proteins [58] and collagen types I, II, and IX [59,60]. These effects have been validated both in vivo by analyzing the juxtaposition and colocalization of these proteins in primate growth plates and in vitro by examining chondrocytes isolated from porcine femoral condyles. Additionally, control experiments with cells were conducted using type II collagen (Col2) scaffolds as 3D cell culture platforms, with nanoclusters composed of Col2-coated antagonistic chondrocyte membrane (CCM) extracts serving as the control group [58,59,60]. Furthermore, a study comparing cartilage tissue blocks from the primary defects and normal lateral condyle regions in 12 patients with KOA further confirmed that COMP can slow down the degradation of cartilage tissue in the extracellular matrix, thereby improving KOA [61].

Dipeptidyl peptidase 4 (DPP4) is a protease expressed in endothelial and epithelial tissues that participates in immune regulation and inflammatory responses via immune cells such as T cells, activated B cells, activated natural killer (NK) cells, and myeloid cells [62]. Experiments on human joint soft tissue cells have demonstrated that activated DPP4 promotes the generation of ROS, triggers oxidative stress responses, activates the NF-κB signaling pathway, induces chronic inflammation, and ultimately leads to chondrocyte senescence and cartilage matrix degradation [63,64]. Furthermore, a cell-based experiment using anti-DPP4 antibodies confirmed these findings by detecting ROS production in endothelial cells and in sorting senescent from non-senescent chondrocyte populations. Therefore, biologically speaking, COMP exhibits a protective role in KOA, whereas plasma DPP4 may contribute to the exacerbation of KOA pathology.

Therefore, from a biological standpoint, the decrease in rQTL-COMP/DPP4 levels may hinder the improvement of KOA, which is inconsistent with the findings of this study. When rQTL-COMP/DPP4 serves as a mediating variable in the influence of C-X-C motif chemokine 9 levels on KOA, the direction of its effect diverges from the total effect. This discrepancy may occur because elevated C-X-C motif chemokine 9 levels reduce the rQTL-COMP/DPP4 ratio, potentially restoring COMP’s protective role while mitigating DPP4’s detrimental effects. This process establishes a compensatory mechanism that amplifies COMP’s protective function, thereby promoting KOA improvement. Although C-X-C motif chemokine 9 levels exhibit pro-inflammatory properties and are positively associated with KOA progression, their regulation of the rQTL-COMP/DPP4 ratio indirectly enhances COMP’s role in KOA improvement, creating a paradoxical co-occurrence of signals.

## 4. Methods and Materials

### 4.1. Data

#### 4.1.1. Exposure Data

Genetic variant data of inflammatory proteins are available for download at https://www.ebi.ac.uk/gwas/ (accessed on 1 October 2024), study accession numbers GCST90274758–GCST90274848. Genome-wide pQTL mapping was conducted for 91 plasma proteins and evaluated using the Olink Target Inflammation Panel in 11 cohorts, encompassing a total of 14,824 participants of European ancestry.

#### 4.1.2. Mediator Data

Variant data of rQTLs are available for download at https://www.ebi.ac.uk/gwas/ (accessed on 1 October 2024), study accession numbers GCST90313126-GCST90315946. Using Olink proteomics data for 1463 proteins from more than 54,000 UK Biobank samples, we identified 4248 associations involving 2821 rQTLs.

#### 4.1.3. Outcome Data

Genetic variant data of KOA are available for download at https://www.ebi.ac.uk/gwas/ (accessed on 1 October 2024), study accession number GCST005813. We obtained the KOA genotype data from genome-wide association studies (GWAS), including 22,347 European individuals, with 4462 cases and 17,885 controls.

### 4.2. Statistics

#### 4.2.1. MR Analysis

##### Forward MR Analysis

**Figure 6:**
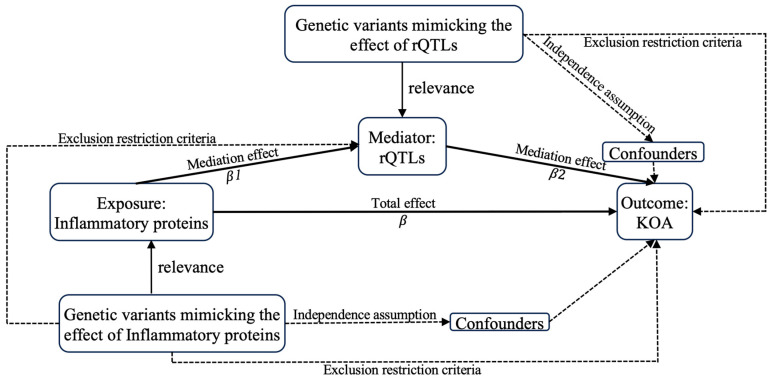
Flowchart of study.

To fulfill the assumptions of relevance, independence, and exclusion restriction in MR (Figure 6), we filtered the exposure and mediator data according to the following steps: (1) Select significant genetic variants (*p* < 0.00001). (2) To ensure that the genetic variants were not in linkage disequilibrium (LD) with each other, we applied an LD threshold of R^2^ < 0.001 and a distance criterion of 10,000 kb. (3) The strength of the instrumental variables (IVs) for inflammatory proteins and rQTLs was assessed using the F-statistic (F = β^2^/SE^2^). IVs with an F-statistic > 10 were selected to reduce bias from weak IVs. (4) To ensure that the same SNPs had consistent alleles in both inflammatory proteins and KOA, as well as rQTLs and KOA, ambiguous alleles were excluded. (5) IVW (inverse variance weighted) is an effective and reliable method for estimating unbiased causal effects. (6) Sensitivity analysis: Heterogeneity was assessed using Cochran’s Q statistic, and horizontal pleiotropy was evaluated via MR-Egger regression. If horizontal pleiotropy was detected, the MR-PRESSO method was employed to identify and remove outliers, followed by a reassessment of pleiotropy. (7) Excluding horizontal pleiotropy (sensitivity analysis *P*_Pleiotropy_ < 0.05), the presence of heterogeneity did not impact the results. The original analysis indicated a significant causal relationship (*P*_IVW_ < 0.05). All analyses were conducted using R software (version 4.4.0) [65,66,67].

##### Reverse MR Analysis

We conducted a second MR analysis using the causal inflammatory proteins and rQTLs derived from forward MR as outcomes, with KOA serving as the exposure. By excluding inflammatory proteins and rQTLs that exhibited reverse causal relationships (reverse MR *P*_IVW_ < 0.05), we ensured that the selected inflammatory proteins and rQTLs represent unidirectional exposures influencing KOA. The method is as follows Section Forward MR Analysis.

#### 4.2.2. Colocalization Analysis

Using the “coloc” R package, we carried out a Bayesian colocalization analysis to explore whether rQTLs and the KOA trait share a common causal variant [68]. The posterior probabilities were evaluated under the following five hypotheses: PPH0: no association with either trait; PPH1: association with the rQTLs trait but not the KOA trait; PPH2: association with the expression of the KOA trait but not the rQTLs trait; PPH3: association with both traits, but with distinct causal variants; PPH4: association with both traits, sharing a common causal variant. If the PPH4 value is greater than 0.75, it is considered compelling evidence of colocalization.

#### 4.2.3. False Discovery Rate Corrected (FDR-Corrected)

**Figure 7:**
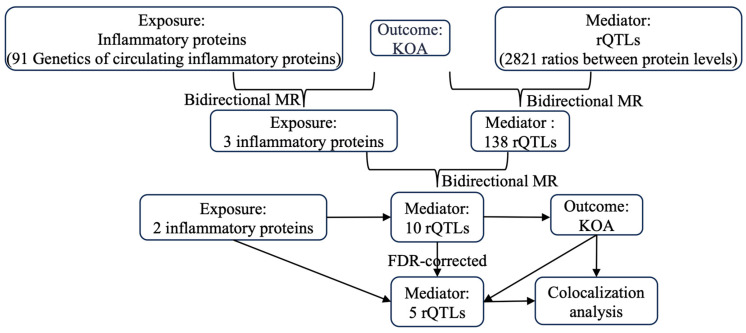
Overview of the study design to identify the intervention targets for KOA.

The results of MR analysis between the 3 rQTLs associated with the C-C motif chemokine 25 levels and KOA, and the results of MR analysis between the 7 rQTLs associated with C-X-C motif chemokine 9 levels and KOA, were corrected for FDR using the Benjamini–Hochberg method [68]. Adjust*P*_IVW_ values were then compared to the predefined threshold of 0.05 to identify significant results (Adjust*P*_IVW_ < 0.05) (Figure 7).

#### 4.2.4. Mediation Analysis

We used the IVW method as our primary approach to estimate the effect of inflammatory proteins on rQTLs (*β*1), the effect of rQTLs on KOA (*β*2), and the total effect of inflammatory proteins on KOA (*β*). The mediated proportion was calculated as follows [69]: (*β*1 × *β*2)/*β* (Figure 6).

## 5. Conclusions

In this study, rQLTs were introduced as mediators to analyze the impact of inflammatory proteins on KOA. Two inflammatory proteins associated with KOA were identified, and changes in their levels were found to induce rQLT alterations, thereby exerting differential effects on KOA. If the direction of the mediating effect of rQLTs in the relationship between inflammatory protein levels and KOA is consistent with the total effect, this suggests that the mediating role of rQLTs in the effect of albumin levels on KOA is plausible.

This study screened 2821 rQLTs and 91 inflammatory proteins, identifying rQLT-ANGPTL3/TFPI as a mediator influencing KOA via the level of T-cell surface glycoprotein CD5. From a biological standpoint, it is plausible that rQLT-ANGPTL3/TFPI serves as a mediator in the effect of T-cell surface glycoprotein CD5 levels on KOA. However, after analysis, the mechanisms of action for the other four rQLTs (rQTL-CPA1/CTRB1, rQTL-HAGH/HBQ1, rQTL-MSRA/P4HB, rQTL-COMP/DPP4) as mediators for KOA remain inconclusive. Therefore, this study concludes that rQLT-ANGPTL3/TFPI is highly likely to serve as a gene target for developing new drugs for KOA and has significant potential as a biomarker for evaluating drug efficacy and prognosis, thereby providing a novel research direction for the innovative diagnosis and treatment of KOA.

### Limitations

Although this study employed MR integrated with protein biological function analysis rather than colocalization analysis, thereby minimizing the impact of confounding factors such as race and environment and ensuring the generalizability of the conclusions to other populations, we identified inconsistencies between some results and the findings from protein biological research during the discussion phase. This may be attributed to the fact that the biological function of a single protein cannot adequately capture the complexity of dynamic interactions among multiple related proteins, leading to an incomplete understanding of protein interactions in KOA. Additionally, compensatory mechanisms, conflicting signals, and the potential underestimation of mediating effects may also contribute to these discrepancies. Therefore, as a preliminary exploratory study, future efforts should focus on refining cell and animal model experiments to elucidate the intricate interaction mechanisms among the relevant proteins. Such advancements will enhance the scientific rigor and biological consistency of the conclusions while improving their broad applicability. Furthermore, this work will provide a robust theoretical foundation for subsequent cell and animal experiments, reduce unnecessary resource use, and guide future research directions.

## Figures and Tables

**Table 1:** Associations of three inflammatory proteins with KOA using bidirectional MR analysis.

Exposure	Outcome	SNPs	OR (95%*CI*)	*P*_IVW_	*P*_Heterogeneity_	*P*_Pleiotropy_	Reverse MR *P*_IVW_
C-C motif chemokine 25 levels	KOA	35	0.904 (0.843–0.969)	0.004	0.387	0.288	0.557
T-cell surface glycoprotein CD5 levels	KOA	31	0.867 (0.760–0.990)	0.035	0.226	0.761	0.858
C-X-C motif chemokine 9 levels	KOA	40	1.150 (1.001–1.320)	0.048	0.128	0.506	0.346

**Table 2:** Reverse MR analysis identified nine rQTLs that were significantly associated with KOA.

Exposure	Outcome	SNPs	OR (95%*CI*)	*P*_IVW_
KOA	rQTL-CASP3/PDGFB	4	1.061 (1.000–1.126)	0.049
KOA	rQTL-CD40/TIMP3	3	1.161 (1.081–1.247)	0.00004
KOA	rQTL-CEBPB/NADK	4	1.068 (1.006–1.135)	0.031
KOA	rQTL-DAG1/PDGFB	4	1.069 (1.008–1.134)	0.027
KOA	rQTL-EREG/TIMP3	3	1.129 (1.050–1.214)	0.001
KOA	rQTL-F2R/PDGFB	4	1.065 (1.004–1.130)	0.037
KOA	rQTL-FXYD5/IRAK1	4	1.074 (1.012–1.140)	0.019
KOA	rQTL-ITGB1/TFPI	4	0.937 (0.884–0.992)	0.026
KOA	rQTL-MVK/SERPINE1	4	1.063 (1.002–1.129)	0.043

**Table 3:** Five rQTLs (Adjust*P*_IVW_ < 0.05) were selected to investigate the mediating role of inflammatory proteins in the progression of KOA.

Exposure	Mediator	Outcome	Mediation Effect *β*1	Mediation Effect *β*2	Total Effect *β*	Mediated Proportion *β*1 × *β*2/*β*
T-cell surface glycoprotein CD5 levels	rQTL-ANGPTL3/TFPI	KOA	−0.084	0.159	−0.143	9.340%
	rQTL-CPA1/CTRB1	KOA	−0.178	−0.095	−0.143	−11.825%
	rQTL-HAGH/HBQ1	KOA	0.097	0.132	−0.143	−8.954%
C-X-C motif chemokine 9 levels	rQTL-COMP/DPP4	KOA	−0.208	0.134	0.140	−19.909%
	rQTL-MSRA/P4HB	KOA	−0.177	−0.230	0.140	29.079%

## Data Availability

The data that support the findings of this study are available from the European Bioinformatics Institute (EBI) public database.
